# Comparison of the effect of non-esterified and esterified astaxanthins on endurance performance in mice

**DOI:** 10.3164/jcbn.17-89

**Published:** 2017-12-27

**Authors:** Wataru Aoi, Takashi Maoka, Ryo Abe, Mayuko Fujishita, Kumi Tominaga

**Affiliations:** 1 Division of Applied Life Sciences, Graduate School of Life and Environmental Science, Kyoto Prefectural University, 1-5 Hangi-Cho, Shimogamo, Sakyo-ku, Kyoto 606-8522, Japan; 2 Research Institute for Production Development, 15 Morimoto-cho, Shimogamo, Sakyo-ku, Kyoto 606-0805, Japan; 3 AstaReal Co., Ltd., 55 Yokohoonji, Kamiichi-machi, Nakaniikawa-gun, Toyama 930-0397, Japan

**Keywords:** astaxanthin, esterified form and non-esterified form, running exercise, energy metabolism, oxidative damage

## Abstract

Astaxanthin, a natural antioxidant, exists in non-esterified and esterified forms. Although it is known that astaxanthin can improve exercise endurance and cause metabolic improvement in skeletal muscle, the effects of the two different forms are unclear. We investigated the effects of the different forms of astaxanthin on endurance in mice. Eight-week-old ICR mice were divided into four groups: control; astaxanthin extracted from *Haematococcus pluvialis* in an esterified form; astaxanthin extracted from *Phaffia rhodozyma* in a non-esterified form; and astaxanthin synthesized chemically in a non-esterified form. After 5 weeks of treatment, each group was divided into sedentary and exercise groups. In the group fed astaxanthin from *Haematococcus*, the running time to exhaustion was longest, and the plasma and tissue concentrations of astaxanthin were significantly higher than those in the other groups. Astaxanthin from *Haematococcus* increased 5'-adenosine monophosphate-activated protein kinase levels in the skeletal muscle. Although the mice in the *Haematococcus* group ran for longer, hexanoyl lysine adduct levels in the skeletal muscle mitochondria were similar in the control and *Haematococcus* groups. Our results suggested that esterified astaxanthin promoted energy production and protected tissues from oxidative damage during exercise owing to its favorable absorption properties, leading to a longer running time.

## Introduction

Astaxanthin (3,3'-dihydroxy-β,β'-carotene-4,4'-dione), a carotenoid, is biosynthesized via a unique metabolic pathway in photosynthetic bacteria, algae and yeasts, although almost all animals lack biosynthetic pathways. It has been known that astaxanthin exists in three different forms related to its two hydroxyl groups: 1) non-esterified form with both hydroxyl groups unmodified, 2) monoesterified form with one hydroxyl group esterified with fatty acid, and 3) diesterified form with both hydroxyl groups esterified with fatty acid. These three forms exist in various ratios depending on the source of synthesis (Table 1).^(1)^ For example, it has been reported that astaxanthin extracted from *Haematococcus* algae consists predominantly of the monoesterified form, while astaxanthin sourced from *Phaffia* yeast is almost all non-esterified. In addition to these extracted naturally occurring materials of natural source, chemically synthesized astaxanthin is available as a purely non-esterified form. Besides the esterified and non-esterified forms, there also exist different types of optical isomers, which occur in various ratios depending on the source.

Astaxanthin is known as a highly potent antioxidant.^(2)^ Reactive oxygen species (ROS) accumulate in the body during exercise, causing oxidative stress.^(3,4)^ Many reports to date provide information on the association of astaxanthin intake with exercise. Our group and other researchers have reported animal studies that astaxanthin decreases oxidative damage to skeletal and cardiac muscles associated with exercise,^(5)^ promotes lipid metabolism during exercise^(6)^ and improvement in endurance performance.^(7)^ Clinical studies also showed improved muscular endurance by astaxanthin intake and better performance in bicycle time trial.^(8,9)^ On the other hand, clinical studies with daily-training athletes showed that astaxanthin intake did not improve their anti-oxidant capacity and performance.^(10,11)^ It is considered that this was due to their endogenous anti-oxidant capacity already being enhanced by their daily training routine. In addition, there are reports that indicate various beneficial effects of astaxanthin, including singlet oxygen quenching activity,^(12)^ antitumor,^(13)^ immunomodulating^(14)^ and anti-inflammatory effects.^(15)^

In the context of multifunctional role of astaxanthin, esterified forms were reported to show higher antioxidant activity than the non-esterified form both in *in vitro* experiments.^(16)^ A study comparing the antitumor effect of esterified and non-esterified forms in a rat model of skin cancer was reported higher activity of the esterified form.^(17)^ Only a limited number of reports are available on the difference of activity between both forms and, in particular, reports comparing exercise performance are not available. Therefore, in this study, we investigated the effect of esterified and non-esterified astaxanthins on endurance performance, and the relationship of energy metabolism and oxidative damage with *in vivo* distribution of astaxanthins after administration to mice.

## Materials and Methods

### Animals and experimental design

The present study complied with the principles and guidelines of the Japanese Council on Animal Care and was also approved by the Committee for Animal Research of Kyoto Prefectural University (KPU280526). ICR mice (8 weeks old) (Oriental Bio Service, Inc., Kyoto, Japan) were acclimatized for 1 week in an air-conditioned (23 ± 2°C) room with a 12-h light/dark cycle (lights on from 7:00 to 19:00). The mice were divided into a control group and 3 astaxanthin groups (HAE, SYN, PHA). Each group had 10 mice and feed with 0.02% (w/w) of astaxanthin as free form was given to all astaxanthin groups. Specifically, astaxanthin extracted from *Haematococcus pluvialis* (AstaReal powder 20F, Fuji Chemical Industries Co., Ltd., Toyama, Japan) to HAE, synthetic astaxanthin (AstaSana, DSM, Heerlen, Limburg, Nederland) to SYN, *Phaffia rhodozyma*-derived astaxanthin (NatuAsta, ASKA Animal Health Co., Ltd., Tokyo, Japan) to PHA were given with feed. The same amount of placebo powder (AstaReal placebo powder 20F) as AstaReal powder 20F was fed to control group. Mice were given *ad libitum* access to food and water for 5 weeks. All mice were accustomed to the treadmill running exercise 3 days a week before the endurance test in order to ensure that all of the mice were equally exposed to stress in the same way. The running speed was gradually increased from 10 m/min to 25 m/min for 5 min. After 5 weeks treatment, each group was divided into 2 groups of 5 animals each: a sedentary group and a running group. Running groups performed treadmill exercise for the assessment of endurance. The running speed was 25 m/min until exhaustion, while the running time to exhaustion was measured. Exhaustion was defined as the inability of a mouse to right itself after being placed on its side. No mice ceased exercise because of injury. Immediately after running, mice were sacrificed by blood withdrawal from the heart. Skeletal muscle, heart and liver were collected after the previous procedure. The tissues of sedentary groups were collected at the same time as the running group. For measurement of the astaxanthin concentration and western blotting, heart and skeletal muscle were homogenized with pestle and mortar under liquid nitrogen, and were subsequently divided in half. All organs were stored at –80°C before measurements.

### Quantitative analysis of astaxanthin in plasma

To 100 µl of plasma samples, 100 µl of trans-β-apo-8'-carotenal (100 ng/ml in acetone; Sigma Aldrich, St. Louis, MO) as the internal standard and 500 µl of butylhydroxytoluene (50 µg/ml in ethanol) were added and stirred. To the mixture, 5 ml of hexane was added followed by further stirring and centrifugation at 3,500 rpm for 10 min. After centrifugation, 4 ml of the supernatant was collected and passed through a membrane filter with a pore size of 0.45 µm. The filtrate was concentrated through evaporation, and the residue was dissolved in 150 µl of acetone and applied to reversed-phase high performance liquid chromatography (HPLC). Standard solutions containing 100 ng/ml astaxanthin (Alexis Biochemicals, Farmingdale, NY) and 100 ng/ml trans-β-Apo-8'-carotenal in acetone was applied to reversed-phase HPLC. By comparing the peak areas obtained above, the plasma astaxanthin concentration was determined.

For HPLC, a Shimadzu LC20A series system (pump, LC-20AD; degasser, DGU-20A5R; auto-sampler, SIL-20AC; column oven, CTO-20AC; detector, SPD-20AV; system controller, CBM-20A; all the components were from Shimadzu Corporation, Kyoto, Japan) was used. A YMC-Carotenoid column (4.6 × 250 mm, 5 µm of particle size, YMC, Kyoto, Japan) was used for the analysis. HPLC analysis used methanol, *tert*-butyl methyl ether and 1% (v/v) phosphate solution as mobile phase A, B and C, respectively, and started at the mobile phase ratio, A:B of 81%:15%. Then, the percentage of mobile phase B changed for elution in a gradient manner to 30% at 15 min and 80% at 23 min, then maintained 80% until 27 min, and returned to the initial condition at 27.1 min. The initial ratio was then maintained until 35 min. The percentage of mobile phase C was maintained at 4% throughout the analysis session. The HPLC analysis was run at 1 ml/min using a column oven at 25°C and an ultraviolet-visible absorption photometer at a detection wavelength of 470 nm.

### Quantitative analysis of astaxanthin in the heart, skeletal muscle and liver

Each organ was extracted twice with acetone. Extracts were combined, and after filtration, the solution was evaporated. Then, the residue was dissolved in diethyl ether/n-hexane (2:8) and submitted to HPLC analysis. HPLC was performed with a Hitachi L-6200 intelligent pump, and an L-4250 UV-VIS detector set at 450 nm, and a 5 µm Cosmosil 5SL-II column (4.6 × 250 nm, Nacalai Tesque, Inc., Kyoto, Japan) with a mobile phase of acetone/n-hexane (2:8) at a flow rate of 1.0 ml/min). The astaxanthin content was quantified relative to a standard sample.

### Western blotting

Proteins were extracted from skeletal muscle and heart tissues of CON and HAE group mice using a lysis buffer (CelLytic MT Cell Lysis Reagent; Sigma Aldrich), and mitochondria were extracted from muscle and heart using mitochondria isolation kit (Abcam plc, Cambridge, UK) according to the manufacturer’s instructions, on all samples and under the same conditions. Equal amounts of protein in the lysates were separated by 10% sodium dodecyl sulfate-polyacrylamide gel electrophoresis (SDS-PAGE), and proteins were then transferred onto nitrocellulose membranes. The blots were incubated with primary antibodies against total-5'-adenosine monophosphate-activated protein kinase (AMPK) α (Cell Signaling Technology, Beverly, MA) and hexanoyl lysine adduct (HEL) (JaICA, Nikken Seil Co., Ltd., Shizuoka, Japan), and reaction products were incubated with horseradish peroxidase (HRP)-conjugated secondary antibodies (GE Healthcare UK Ltd., Buckinghamshire, UK), followed by detection via chemiluminescence (ImmunoStar Zeta; Wako Pure Chemical Industries, Ltd., Osaka, Japan, or Chemi-Lumi One Super; Nacalai Tesque). Band densities were measured using Image J software (NIH, Research Service Branch).

### Statistical analysis

All data were reported as the mean ± SD. Outliers were detected using the Smirnov-Grubbs test and removed. Differences between the groups were evaluated using one-way or two-way analysis of variance (ANOVA) or Student’s *t* test. If ANOVA indicated a significant difference, the Tukey-Kramer post-hoc test was used to determine the significance of the differences between the means. Values of *p*<0.05 were considered to indicate statistical significance.

## Results

### Body weight

There were no significant differences in body weight in all experiments groups (CON: 44.8 ± 5.9 g; HAE: 43.5 ± 3.7 g; SYN: 43.6 ± 4.0 g; PHA: 42.5 ± 4.5 g).

### Running time to exhaustion

**Fig. 1:**
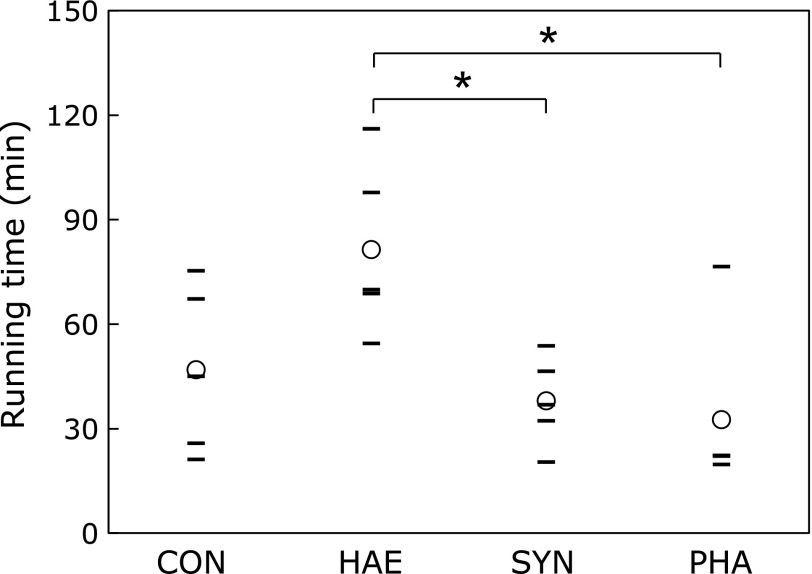
Running time to exhaustion. Exercise groups performed treadmill running at 25 m/min after 5 weeks, and running time to exhaustion was measured. Circles indicate mean values and lines indicate individual values. *n* = 5. *Significant differences between groups at the *p*<0.05 level by using Tukey-Kramer test. CON, control group; HAE, *Haematococcus* astaxanthin group; SYN, synthetic astaxanthin group; PHA, *Phaffia* astaxanthin group.

The results from the treadmill running test are shown in Fig. 1. There was no significant difference in running time comparing HAE, SYN, PHA and CON (*p* = 0.106 vs HAE, *p* = 0.919 vs SYN, *p* = 0.741 vs PHA). However, the running time was significantly longer in the HAE group than in the SYN (*p* = 0.032) and PHA groups (*p* = 0.015).

### Concentration of astaxanthin in plasma and tissues

The concentration of astaxanthin in plasma, skeletal muscle, heart and liver are shown in Table 2. Because ingested esterified astaxanthin has been known to be detected as non-esterified form in plasma and tissues in mammals,^(18)^ the concentrations of non-esterified astaxanthin were determined. For all examined tissues, the concentrations of astaxanthin were significantly higher in the HAE group than in the control, SYN, and PHA groups. In the liver, the concentrations of astaxanthin in the PHA group were significantly higher than those in the control group in the sedentary condition. The concentrations of astaxanthin in tissues were not significantly different between sedentary and running animals within each group.

### AMPK contents in skeletal muscle

**Fig. 2:**
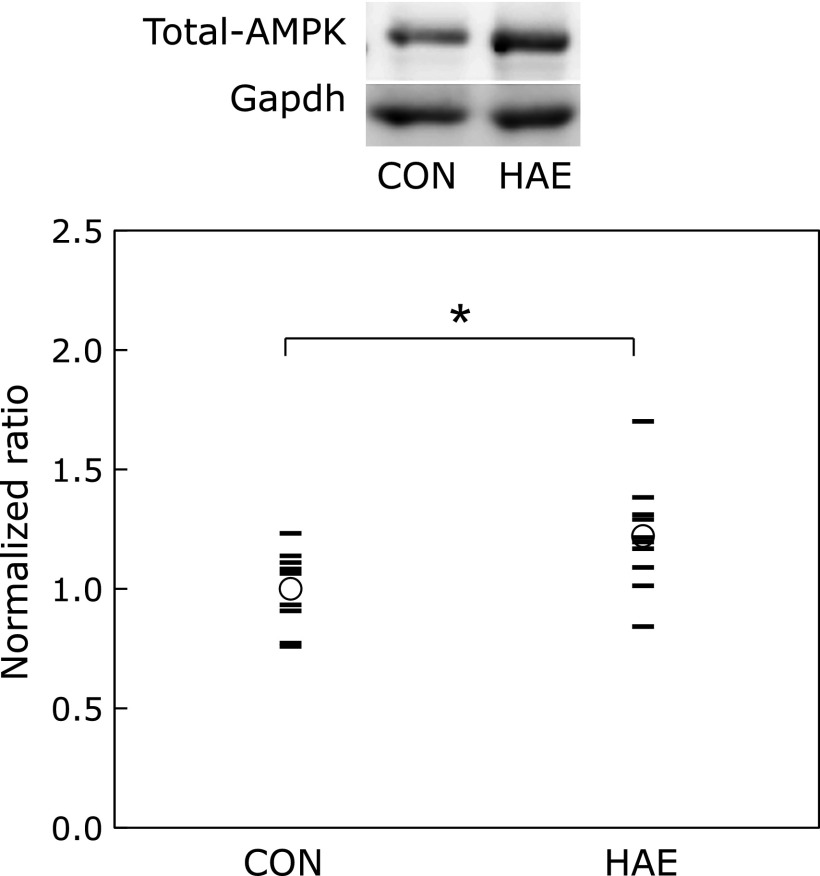
The effect of astaxanthin on total AMPK level in skeletal muscle. Circles indicate mean values and lines indicate individual values. *n* = 9–10. *Significant differences at the level of *p*<0.05 vs control group by using Student’s *t* test. CON, control group; HAE, *Haematococcus* astaxanthin group; AMPK, 5'-adenosine monophosphate-activated protein kinase; Gapdh, glyceraldehyde 3-phosphate dehydrogenase.

As shown in previous reports,^(19,20)^ total AMPK level does not change in response to a single bout of exercise for approximately 1 h. Thus, we analyzed the data from all animals combined, sedentary or running, for the control group and the HAE group. The level of total AMPK in skeletal muscle was significantly augmented in the HAE group than in the control group (Fig. 2).

### Oxidative damage in skeletal muscle and heart

**Fig. 3:**
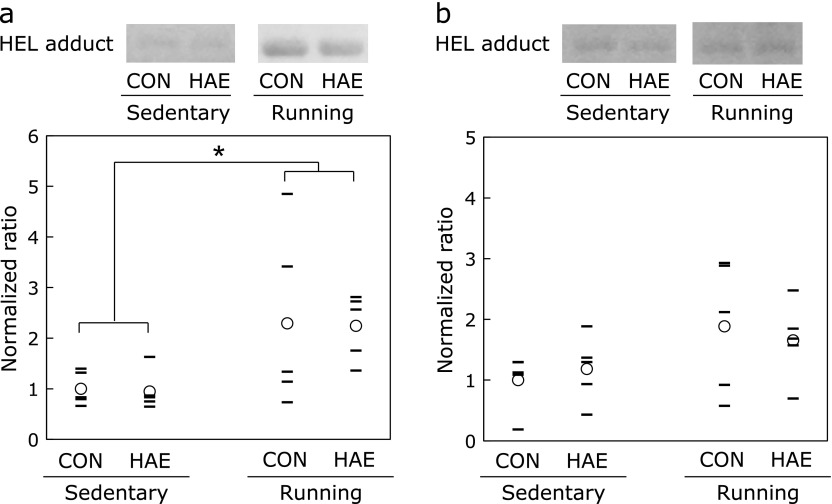
HEL-modified protein levels in (a) skeletal muscle and (b) heart. Circles indicate mean values and lines indicate individual values. *n* = 5. *Significant differences between groups at the *p*<0.05 level were evaluated using Tukey-Kramer test. CON, control group; HAE, *Haematococcus* astaxanthin group; HEL, hexanoyl lysine.

 The levels of HEL in the mitochondria of the skeletal muscle are shown in Fig. 3a. Based on two-way ANOVA, the level of HEL in the running groups was significantly higher than that in the sedentary groups. However, no significant differences were observed in the levels of HEL between the control group and the HAE group. There was no significant interaction effect. The levels of HEL in the heart mitochondria are shown in Fig. 3b. Based on two-way ANOVA, the factors of exercise and treatment had no significant effect on the levels of HEL in heart mitochondria. There was no significant interaction effect.

## Discussion

This is the first study to compare the effect of astaxanthin on exercise performance of mice administered non-esterified and esterified astaxanthin. The mice were administered astaxanthin isolated from *Haematococcus pluvialis*, *Phaffia rhodozyma*, or obtained synthetically for 5 weeks and subjected to an assessment of their running time to exhaustion via a treadmill running test and an analysis of astaxanthin concentrations in each tissue. The animals in the HAE group ran for the longest time in 4 groups, along with higher tissue astaxanthin levels than other groups. Generally, the *in vivo* absorption of carotenoids has been known to be better in the esterified form than in the non-esterified form.^(21)^ For example, 3*R*,3'*R*-zeaxanthin dipalmitate had higher bioavailability than non-esterified form.^(22)^ In addition, astaxanthin applied with molecular delivery technologies, such as liposomes encapsulating, also could have potent scavenging effects against various ROS and ultraviolet-induced skin damage could be prevented more suitably by transdermal administration of liposomal astaxanthin than non-liposomal astaxanthin.^(23–25)^ In the present study, in the group administered esterified astaxanthin, the HAE group, the tissue astaxanthin level was higher than those in the groups administered non-esterified astaxanthin, the SYN and PHA groups. Furthermore, the HAE group showed a longer running time than that shown by the other groups, suggesting that accumulation of astaxanthin in the tissue might contribute to endurance. Therefore, to elucidate the mechanism underlying astaxanthin’s activity, we investigated the energy metabolism and oxidative damage in the tissue in the HAE group, in which the accumulation of astaxanthin in the tissue was greater than that in the other groups.

In our efforts to explain the longer running time of the animals in the HAE group, we first focused on AMPK as a metabolic sensor in mammalian cells. AMPK plays an important role in the regulation of both carbohydrate and lipid metabolism. Specifically, AMPK activation induces the translocation of the glucose transporter-4 in an insulin-independent manner, which leads to the stimulation of cellular glucose uptake.^(26)^ AMPK also inhibits fatty acid synthesis via the suppression of acetyl coenzyme A carboxylase (ACC) and hydroxymethylglutaryl coenzyme A (HMG-CoA) reductase activities, which are required for fatty acid and cholesterol synthesis, and further stimulates the β-oxidation of fatty acids.^(27)^ In addition, AMPK is known to increase mitochondrial biosynthesis and activity via the transcription cofactor, peroxisome proliferator-activated receptor-γ coactivator-1α.^(28)^ The most prominent role of mitochondria is the energy production via the citric acid cycle and the electron transport system. The stimulation of mitochondrial biogenesis results in active energy synthesis, supporting a continuous energy supply that allows long-term exercise. In the present study, the intake of esterified astaxanthin significantly increased AMPK levels; that is, animals administered esterified astaxanthin exhibited a longer running time owing to the activation of energy metabolism.

ROS are generated in the body during exercise and can cause oxidative stress.^(3,4)^ HEL is a lipid-lysine adduct used as an oxidation marker that reflects the initial phase of lipid peroxidation caused by oxidative stress.^(29)^ It has been reported that HEL increases with various causes of oxidative stress. For example, cisplatin of anticancer drug and aristolochic acid of plant alkaloid are known to enhance oxidative stress.^(30,31)^ In addition, HEL is also increased in skeletal muscle by aerobic exercise.^(32)^ Exercise-induced oxidative damage is known to be elevated with duration time.^(3,4,33)^ The comparison of the levels of HEL in skeletal muscle mitochondria between sedentary and running animals in the present study demonstrated that animals experienced oxidative stress after running. On the other hand, there was no significant difference when comparing running time among the 4 groups but when comparing that in the 2 groups of CON and HAE, positive effect of esterified astaxanthin was seen (*p* = 0.057, Student’s *t* test). Furthermore, despite the difference in running time, there was no significant difference in HEL between the exercise groups of CON and HAE (*p*>0.05). Therefore it seems that intake of esterified astaxanthin can inhibit the HEL increase due to a running exercise. A number of reports have demonstrated that astaxanthin has superior antioxidant potency and participates in the protection of tissues from oxidative damage *in vivo*.^(34–36)^ We have also reported that astaxanthin intake inhibited the oxidative modification of carnitine palmitoyltransferase I (CPT-I) on the mitochondrial outer membrane of skeletal muscle.^(6)^ On the other hand, our previous study showed that astaxanthin intake enhances the biosynthesis of mitochondria, a result which we consider to be due to the improvement of aerobic metabolism in mitochondria.^(37)^

In contrast, no significant differences were observed in the levels of HEL in the heart mitochondria between the sedentary and running groups and between the control and HAE groups; an interaction effect between exercise and treatment was not observed. The oxidative stress level during exercise is known to be lower in the heart than in skeletal muscle;^(4)^ therefore, the experimental conditions in the present study might have resulted in less oxidative stress in the heart, which resulted in no significant difference in oxidative damage between running and sedentary animals. However, in our previous study, we measured 4-hydroxynonenal-modified protein and 8-hydroxy-2'-deoxyguanosine as markers of oxidative damage to lipid and DNA, respectively. These markers were significantly decreased by astaxanthin intake.^(5)^ In the present study, though the HAE group ran for a longer time than the control group, the level of oxidative damage in the HAE group was as low as that in the control group. This may indicate that HAE group showed an increased heart astaxanthin level, which helped to protect the heart from oxidative damage caused by a longer running time compared to that of the control group; however further studies are needed.

Other reported effects of astaxanthin intake include inhibition of blood lactic acid elevation.^(38)^ Generation of lactic acid lowers pH and hydrogen ions inhibit skeletal muscle contraction. However, astaxanthin prevents oxidative modification of CPT-I on the mitochondrial membrane, accelerates lipid utilization as an energy substrate, and inhibits the elevation of lactic acid as a carbohydrate metabolite.^(6)^ In the present study, intake of esterified astaxanthin prevented oxidative damage in mitochondria of skeletal muscle, also suggesting possible suppression of lactic acid generation and eventual extension of the running time to exhaustion.

It is also known that peripheral transport of blood oxygen improves muscular endurance.^(39)^ There are other reports, indicating sufficient astaxanthin dosage enables the transfer of astaxanthin from plasma to erythrocytes and astaxanthin shows an inhibitory effect on phospholipid peroxidation in erythrocytes,^(40–42)^ improves erythrocyte deformability via antioxidant effect,^(43)^ and has a vasodilating effect mediated by nitric oxide.^(44)^ These reports indicate that astaxanthin suppresses oxidative damage in erythrocytes, stimulates blood flow properties, and helps transport of blood constituents, such as erythrocytes, to peripheral tissues. Because astaxanthin functions in various aspects, the effect of esterified astaxanthin intake on exercise performance likely involves more diverse mechanisms than mentioned above, warranting further investigation.

In conclusion, our present study compared the effect of non-esterified vs esterified astaxanthin intake on exercise performance and distribution in the body. *Haematococcus* algae-derived, predominantly esterified astaxanthin material had the greatest effect on exercise performance. This is likely attributed to the difference in the absorption between non-esterified and esterified forms; the long running time by mice in HAE may have been due to increased AMPK and sustained HEL level in skeletal muscle and heart during the treadmill running test. Given that astaxanthin is associated with various effects, additional mechanisms may be involved and further research may be required to elucidate the current findings.

## Figures and Tables

**Table 1:** Chemical differences of astaxanthin

Source	Optical isomers	3,3'-OH group modification
*Haematococcus pluvialis*	3*S*, 3'*S*	Monoesterified form rich
Synthetic	3*S*, 3'*S*:meso:3*R*, 3'*R* = 1:2:1	Free form
*Phaffia rhodozyma*	3*R*, 3'*R*	Free form

**Table 2:** Astaxanthin concentration in tissues

Group		Plasma (ng/ml)	Skeletal muscle (ng/g)	Heart (ng/g)	Liver (ng/g)
CON	Sedentary	n.d.	n.d.	n.d.	n.d.
	Running	n.d.	n.d.	n.d.	n.d.
HAE	Sedentary	294.5 ± 114.6*^,†,§^	268.2 ± 104.9*^,†,§^	1,379.2 ± 470.5*^,†,§^	2,559.4 ± 530.9*^,†,§^
	Running	277.5 ± 97.4*^,†,§^	318.0 ± 165.6*^,†,§^	1,033.3 ± 322.1*^,†,§^	2,682.5 ± 812.4*^,†,§^
SYN	Sedentary	1.4 ± 0.7	10.8 ± 3.9	71.6 ± 50.2	93.3 ± 31.6
	Running	1.1 ± 0.3	21.0 ± 11.7	54.9 ± 25.4	50.3 ± 34.2
PHA	Sedentary	53.8 ± 21.0	54.9 ± 16.6	164.8 ± 62.3	588.0 ± 236.0*
	Running	50.4 ± 14.7	78.4 ± 40.6	205.3 ± 52.3	463.5 ± 258.2

Values are means ± SD obtained from 4–5 mice. *^,†,§^Significant differences at the level of *p*<0.05 vs control group (*), synthetic astaxanthin group (^†^), *Phaffia* astaxanthin group (^§^) in sedentary or running group by using Tukey-Kramer test. No significant differences at the *p*<0.05 level of running vs sedentary groups were detected using Student’s *t* test. n.d., not detected; CON, control group; HAE, *Haematococcus* astaxanthin group; SYN, synthetic astaxanthin group; PHA, *Phaffia* astaxanthin group.

## Abbreviations

ACC: acetyl coenzyme A carboxylase
AMPK: 5'-adenosine monophosphate-activated protein kinase
ANOVA: analysis of variance
CPT-I: carnitine palmitoyltransferase I
HEL: hexanoyl lysine adduct
HMG-CoA: hydroxymethylglutaryl coenzyme A
HPLC: high performance liquid chromatography
HRP: horseradish peroxidase
ROS: reactive oxygen species
SDS-PAGE: sodium dodecyl sulfate-polyacrylamide gel electrophoresis

## References

[1] Ambati RR, Phang SM, Ravi S, Aswathanarayana RG (2014). Astaxanthin: sources, extraction, stability, biological activities and its commercial applications--a review. Mar Drugs.

[2] Di Mascio P, Devasagayam TP, Kaiser S, Sies H (1990). Carotenoids, tocopherols and thiols as biological singlet molecular oxygen quenchers. Biochem Soc Trans.

[3] Bejma J, Ji LL (1999). Aging and acute exercise enhance free radical generation in rat skeletal muscle. J Appl Physiol (1985).

[4] Ji LL (1995). Exercise and oxidative stress: role of the cellular antioxidant systems. Exerc Sport Sci Rev.

[5] Aoi W, Naito Y, Sakuma K (2003). Astaxanthin limits exercise-induced skeletal and cardiac muscle damage in mice. Antioxid Redox Signal.

[6] Aoi W, Naito Y, Takanami Y (2008). Astaxanthin improves muscle lipid metabolism in exercise via inhibitory effect of oxidative CPT I modification. Biochem Biophys Res Commun.

[7] Ikeuchi M, Koyama T, Takahashi J, Yazawa K (2006). Effects of astaxanthin supplementation on exercise-induced fatigue in mice. Biol Pharm Bull.

[8] Malmsten CL, Lignell Å (2008). Dietary supplementation with astaxanthin-rich algal meal improves strength endurance - a double blind placebo controlled study on male students. Carotenoid Sci.

[9] Earnest CP, Lupo M, White KM, Church TS (2011). Effect of astaxanthin on cycling time trial performance. Int J Sports Med.

[10] Klinkenberg LJ, Res PT, Haenen GR (2013). Effect of antioxidant supplementation on exercise-induced cardiac troponin release in cyclists: a randomized trial. PLoS One.

[11] Res PT, Cermak NM, Stinkens R (2013). Astaxanthin supplementation does not augment fat use or improve endurance performance. Med Sci Sports Exerc.

[12] Nishida Y, Yamashita E, Miki W (2007). Quenching activities of common hydrophilic and lipophilic antioxidants against singlet oxygen using chemiluminescence detection system. Carotenoid Sci.

[13] Chew BP, Park JS, Wong MW, Wong TS (1999). A comparison of the anticancer activities of dietary β-carotene, canthaxanthin and astaxanthin in mice *in vivo*. Anticancer Res.

[14] Jyonouchi H, Zhang L, Gross M, Tomita Y (1994). Immunomodulating actions of carotenoids: enhancement of *in vivo* and *in vitro* antibody production to T-dependent antigens. Nutr Cancer.

[15] Bennedsen M, Wang X, Willén R, Wadström T, Andersen LP (1999). Treatment of *H. pylori* infected mice with antioxidant astaxanthin reduces gastric inflammation, bacterial load and modulates cytokine release by splenocytes. Immunol Lett.

[16] Régnier P, Bastias J, Rodriguez-Ruiz V (2015). Astaxanthin from *Haematococcus pluvialis* prevents oxidative stress on human endothelial cells without toxicity. Mar Drugs.

[17] Rao AR, Sindhuja HN, Dharmesh SM, Sankar KU, Sarada R, Ravishankar GA (2013). Effective inhibition of skin cancer, tyrosinase, and antioxidative properties by astaxanthin and astaxanthin esters from the green alga *Haematococcus pluvialis*. J Agric Food Chem.

[18] Showalter LA, Weinman SA, Østerlie M, Lockwood SF (2004). Plasma appearance and tissue accumulation of non-esterified, free astaxanthin in C57BL/6 mice after oral dosing of a disodium disuccinate diester of astaxanthin (*Heptax*^TM^).. Comp Biochem Physiol C Toxicol Pharmacol.

[19] Morissette MP, Susser SE, Stammers AN (2014). Differential regulation of the fiber type-specific gene expression of the sarcoplasmic reticulum calcium-ATPase isoforms induced by exercise training. J Appl Physiol (1985)..

[20] Cacicedo JM, Gauthier MS, Lebrasseur NK, Jasuja R, Ruderman NB, Ido Y (2011). Acute exercise acitivates AMPK and eNOS in the mouse aorta. Am J Physiol Heart Circ Physiol.

[21] Mortensen A, Britton G, Liaaen-Jensen S, Pfander H (2009). Supplements. Carotenoids, Volume 5: Nutrition and Health (1st ed.)..

[22] Breithaupt DE, Weller P, Wolters M, Hahn A (2004). Comparison of plasma responses in human subjects after the ingestion of 3R,3R'-zeaxanthin dipalmitate from wolfberry (*Lycium barbarum*) and non-esterified 3R,3R'-zeaxanthin using chiral high-performance liquid chromatography. Br J Nutr.

[23] Kamezaki C, Nakashima A, Yamada A (2016). Synergistic antioxidative effect of astaxanthin and tocotrienol by co-encapsulated in liposomes. J Clin Biochem Nutr.

[24] Hama S, Uenishi S, Yamada A (2012). Scavenging of hydroxyl radical in aqueous solution by astaxanthin encapsulated in liposomes. Biol Pharm Bull.

[25] Hama S, Takahashi K, Inai Y (2012). Protective effects of topical application of a poorly soluble antioxidant astaxanthin liposomal formulation on ultraviolet-induced skin damage. J Pharm Sci.

[26] Yamaguchi S, Katahira H, Ozawa S (2005). Activators of AMP-activated protein kinase enhance GLUT4 translocation and its glucose transport activity in 3T3-L1 adipocytes. Am J Physiol Endocrinol Metab.

[27] Motoshima H, Goldstein BJ, Igata M, Araki E (2006). AMPK and cell proliferation - AMPK as a therapeutic target for atherosclerosis and cancer. J Physiol.

[28] Reznick RM, Shulman GI (2006). The role of AMP-activated protein kinase in mitochondrial biogenesis. J Physiol.

[29] Kato Y, Mori Y, Makino Y (1999). Formation of *N*^ε^-(hexanonyl)lysine in protein exposed to lipid hydroperoxide. A plausible marker for lipid hydroperoxide-derived protein modification. J Biol Chem.

[30] Sugiyama A, Sun J, Nishinohara M (2011). Expressions of lipid oxidation markers, N^ε^-hexanoyl lysine and acrolein in cisplatin-induced nephrotoxicity in rats. J Vet Med Sci.

[31] Matsui K, Kamijo-Ikemorif A, Sugaya T, Yasuda T, Kimura K (2011). Renal liver-type fatty acid binding protein (L-FABP) attenuates acute kidney injury in aristolochic acid nephrotoxicity. Am J Pathol.

[32] Kato Y, Miyake Y, Yamamoto K (2000). Preparation of a monoclonal antibody to *N*^ε^-(hexanonyl)lysine: application to the evaluation of protective effects of flavonoid supplementation against exercise-induced oxidative stress in rat skeletal muscle. Biochem Biophys Res Commun.

[33] Bloomer RJ, Makowski G (2008). Effect of Exercise on Oxidative Stress Biomarker. Advances in Clinical Chemistry, Volume 46 (1st ed.)..

[34] Augusti PR, Conterato GM, Somacal S (2008). Effect of astaxanthin on kidney function impairment and oxidative stress induced by mercuric chloride in rats. Food Chem Toxicol.

[35] Tominaga K, Hongo N, Karato M, Yamashita E (2012). Cosmtic benefits of astaxanthin on humans subjects. Acta Biochim Pol.

[36] Kuraji M, Matsuno T, Satoh T (2016). Astaxanthin affects oxidative stress and hyposalivation in aging mice. J Clin Biochem Nutr.

[37] Liu PH, Aoi W, Takami M (2014). The astaxanthin-induced improvement in lipid metabolism during exercise is mediated by a PGC-1α increase in skeletal muscle. J Clin Biochem Nutr.

[38] Sawaki K, Yoshigi H, Aoki K (2002). Sports performance benefits from taking natural astaxanthin characterized by visual acuity and muscle fatigue improvement in humans. J Clin Ther Med.

[39] Mairbäurl H (2013). Red blood cells in sports: effects of exercise and training on oxygen supply by red blood cells. Front Physiol.

[40] Nakagawa K, Kiko T, Miyazawa T (2011). Antioxidant effect of astaxanthin on phospholipid peroxidation in human erythrocytes. Br J Nutr.

[41] Miyazawa T, Nakagawa K, Kimura F, Satoh A, Miyazawa T (2011). Erythrocytes carotenoids after astaxanthin supplementation in middle-aged and senior Japanese subjects. J Oleo Sci.

[42] Miyazawa T, Nakagawa K, Kimura F, Satoh A, Miyazawa T (2011). Plasma carotenoid concentrations before and after supplementation with astaxanthin in middle-aged and senior subjects. Biosci Biotechnol Biochem.

[43] Miyawaki H, Takahashi J, Tsukahara H, Takehara I (2008). Effects of astaxanthin on human blood rheology. J Clin Biochem Nutr.

[44] Hussein G, Nakamura M, Zhao Q (2005). Antihypertensive and neuroprotective effects of astaxanthin in experimental animals. Biol Pharm Bull.

